# Insights Into AI-Enabled Early Diagnosis of Oral Cancer: A Scoping Review

**DOI:** 10.7759/cureus.88407

**Published:** 2025-07-21

**Authors:** Mamata Kamat, Uma V Datar, Varsha Vimal Kumar

**Affiliations:** 1 Oral Pathology and Microbiology, Bharati Vidyapeeth (Deemed to be University) Dental College and Hospital, Sangli, IND; 2 Oral Pathology and Microbiology, RajaRajeswari Dental College and Hospital, Bangalore, IND

**Keywords:** artificial intelligence, early detection, oral cancer, scoping review, early diagnosis

## Abstract

Oral cancer (OC) remains a significant global health burden, with early detection being critical to improve prognosis and survival rates. Hence, early assessment is the primary challenge in improving OC outcomes due to gaps in specialist referrals and early diagnosis. Recently, artificial intelligence (AI) has emerged as a promising tool to enhance the early detection of oral potentially malignant disorders (OPMDs) and OC. We aimed to assess the various AI techniques for early OC diagnosis by searching PubMed for articles published between January 2016 and May 2025 using terms like "artificial intelligence", "deep learning", "machine learning", and "oral cancer". Following the Preferred Reporting Items for Systematic reviews and Meta-Analyses extension for Scoping Reviews (PRISMA-ScR) guidelines, a comprehensive literature search was conducted. Of 88 articles retrieved, 28 met the inclusion criteria. Most studies originated from Southeast Asia and employed AI methods such as convolutional neural networks (CNNs), deep CNNs, artificial neural networks (ANNs), random forests, and decision trees. Standard data inputs included photographic and mobile images, with cytology and radiographic images also used. Deep CNNs showed the highest performance concerning sensitivity, specificity, and accuracy. Despite variability in techniques and datasets, overall diagnostic performance was promising. The study indicates that AI tools offer a strong potential for enhancing early diagnosis, particularly in low-resource settings.

## Introduction and background

According to GLOBACCON 2022, the incidence of lip and oral cavity cancer is projected to surge from 390,000 in 2022 to 600,000 by 2045, with an expected rise in deaths from 188,000 to 294,000 [1]. In India, oral cancer (OC) is among the leading cancers, with over 135,000 new cases reported annually, contributing to nearly one-third of the global burden. OC imposes a significant health burden. Even with advanced diagnostic and therapeutic procedures, the five-year survival rate has not improved significantly. Hence, OC screening is critical to improve prognosis and survival rates. However, a lack of widespread availability and interobserver variability challenges the currently available screening approaches. The current poor surveillance at local care centres, leading to delayed referrals to specialists, signifies the critical need for early diagnosis in the effective management of OC [2]. In oral oncology, artificial intelligence (AI) applications are being developed to improve the accuracy, speed, and accessibility of early detection, particularly through non-invasive methods.

Several recent studies have demonstrated promising results across various data modalities and algorithmic frameworks and highlighted the potential for AI in the early diagnosis of oral potentially malignant disorders and OC, opening up promising new avenues for screening [1-31].

These studies highlight how AI increasingly becomes part of the OC care journey, assisting with early, noninvasive screening and real-time lesion detection to predict deeper molecular risks. Early recognition of OC reduces mortality and improves therapeutic outcomes. Integrating AI in early diagnosis can enhance disease outcomes related to recurrence and survival. The present scoping review highlights the current range of AI applications for the early diagnosis of OC, emphasizing the different models used, the types of data they rely on, and their performance.

## Review

Methodology

Search Strategy

A comprehensive search of the PubMed database was conducted to identify articles related to AI and OC diagnoses. The search was limited to human studies in English published between January 2016 and May 2025. The search terms included (artificial intelligence OR deep learning OR machine learning) AND (oral cancer) AND (diagnosis). Additional relevant studies were included to ensure the review was as comprehensive as possible, using a combination of MeSH terms, keywords, and manual reference checks (Table 1).

**Table 1:** MeSH (Medical Subject Headings) terms used in the review

	MeSH terms used	Keywords (free text)
Artificial intelligence	"Artificial Intelligence"[MeSH]	artificial intelligence, machine learning, deep learning
Oral cancer	"Mouth Neoplasms"[MeSH]	oral cancer
Diagnosis	"Diagnosis"[MeSH]	diagnosis, detection, screening

Inclusion and Exclusion Criteria

Articles were selected based on clear and specific inclusion and exclusion criteria. Inclusion criteria were full-text articles of original research indicating the use of AI for diagnosing OC and papers with sufficient data related to the source of data input, AI methods used, and performance, among others. Exclusion criteria were reports on the use of AI in prognosis and treatment, duplicate articles, and irrelevant articles like narrative reviews and opinions. Bibliographies of relevant articles were also reviewed.

PICOS criteria applied in the review are as follows: Population (P): patients with OC; Intervention (I): application of AI techniques (e.g., machine learning, deep learning (DL)) for diagnostic purposes; Comparison (C): not applicable; Outcome (O): Diagnostic performance, accuracy, sensitivity, and specificity of AI methods in the early detection of OC; Study Design (S): original human studies (retrospective, prospective, observational, or experimental) published between January 2016 and May 2025 in English.

Selection and Data Plotting

Two authors (MK and UD) independently evaluated the full-text articles for eligibility. Then, detailed data were extracted. The disagreements were resolved by consensus. The extracted data were tabulated based on study characteristics (author, year, etc.), modality (source of input data), AI method used, and diagnostic ability.

Critical Appraisal

A formal risk-of-bias assessment was not conducted in this scoping review, as the primary aim was to comprehensively chart the breadth and diversity of existing research on AI in OC diagnosis, rather than to generate pooled effect estimates through quantitative synthesis. However, evidence quality was considered during synthesis, with greater weight given to studies with robust design, particularly those using validated AI models, larger datasets, and advanced imaging such as histopathology, clinical photos, and CT scans for OC diagnosis.

Results

**Figure 1:**
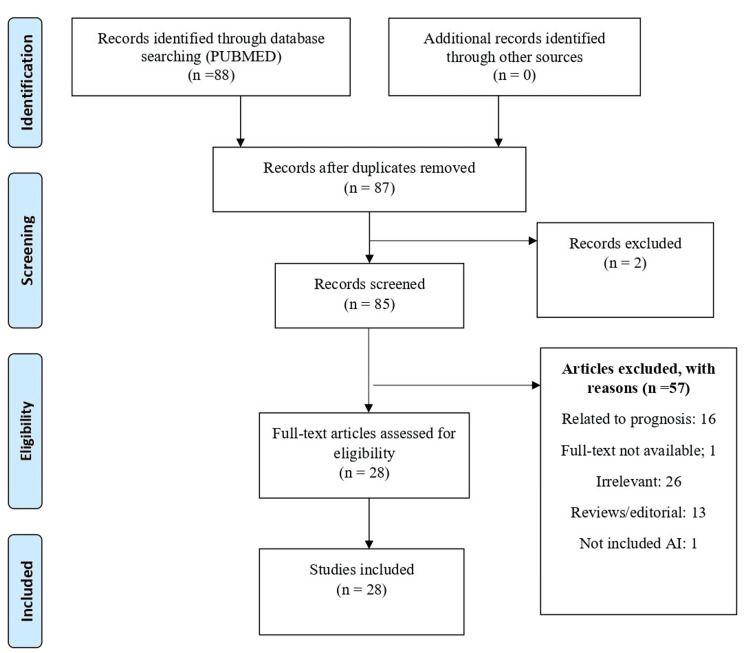
Summary of search strategy

Eighty-eight articles were initially retrieved. After initial analysis, non-relevant and duplicate items were excluded. Finally, 28 full-text papers were included, which met the inclusion criteria [4-31]. The search strategy is summarized in Figure 1.

Most studies originated in technology departments (n = 4) [5,21,22,28]. They often collaborated with OC specialists (n = 24) [4,6-20,23-27,29,30,31]. Data input primarily came from biopsy samples and clinical images (Table 2). Table 3 summarizes the findings of the included studies.

**Table 2:** Source of data input used in the studies

Source of data input	No. of samples/images
Photographic images [6,9,10,17,18,26]	22–700 images
Mobile-based images [8,12,30]	7970 images
Radiographic images [20]	50–70 images
Cytology [24,31]	200–700 samples
Saliva-based samples [13,19]	30–50 samples
Biopsy/histopathology	40–176 images
[4,5,15,16,22,28,29]	
Exhaled breath [7]	105 samples
Clinical images/data [11,14,21,23,25,27]	Variable

**Table 3:** Summary of the findings of the studies Note: “—” denotes that the respective value (accuracy, sensitivity, or specificity) was not provided in the original publication. AI: artificial intelligence, ML: machine learning, DL: deep learning, CNN: convolutional neural network, SVM: support vector machine, GLCM: gray-level co-occurrence matrix, LDA: linear discriminant analysis, KNN: k-nearest neighbors, GSO-ESNN: gravitational search optimized echo state neural network, FDL: feature discriminant loss, MIL: multiple instance learning, CT: computed tomography, CBCT: cone beam computed tomography, OPG: orthopantomogram, VOC: volatile organic compounds, DICOM: digital imaging and communications in medicine, OSCC: oral squamous cell carcinoma, OPMDs: oral potentially malignant disorders, PMOLs: potentially malignant oral lesions, FTIR: Fourier transform infrared spectroscopy, VGG16 / VGG19: Visual Geometry Group Network (with 16 or 19 layers), ResNet: residual network, DenseNet121: densely connected convolutional neural network (121 layers), Faster R-CNN: faster region-based convolutional neural network

Sr. no.	Author	Modality	AI method	Key findings	Accuracy (%)	Sensitivity (%)	Specificity (%)	Study design
1	Kim et al. [4]	Transcriptome data	Subnetwork rep. learning	Identified biomarkers predictive of lymph node metastasis in early oral cancer	High	—	—	Retrospective cohort (computational transcriptome analysis)
2	Caughlin et al. [5]	Fluorescence lifetime imaging	End-to-end neural network	Improved diagnostic sensitivity over SVM	—	+6.5–8.3 over SVM	—	Prospective technical validation (in vivo diagnostic test)
3	Song et al. [6]	Clinical photographic images	CNN	Addressed data imbalance; high classification performance	88.8	88.2	97	Experimental deep learning validation study
4	Mentel et al. [7]	Breath samples	Machine learning	VOCs predicted OSCC with high accuracy	86–90	—	—	Prospective case-control study
5	Lin et al. [8]	Smartphone-based images	Deep learning	Feasibility of smartphone-AI diagnosis	—	—	—	Retrospective diagnostic validation study
6	Narayanan et al. [9]	Multispectral imaging	Cloud-based ML	Real-time screening and biopsy guidance for OPMDs	—	—	—	Prospective pilot diagnostic study
7	Warin et al. [10]	Photographic images	DenseNet121, faster R-CNN	99% precision; 98.75% sensitivity in classification	99	98.75	100	Retrospective diagnostic validation study
8	Alhazmi et al. [11]	Clinical and epidemiological data	Artificial neural network	78.95% accuracy, 85.71% sensitivity, 60% specificity in cancer risk prediction	78.95	85.71	60	Retrospective diagnostic/prognostic prediction study
9	Song et al. [12]	Mobile imaging	CNN	Mobile-based oral cancer classification system	—	—	—	Prospective diagnostic validation study
10	Monedeiro et al. [13]	Salivary VOCs	Artificial neural network	90% accuracy, 100% sensitivity and specificity	90	100	100	Retrospective observational diagnostic validation study
11	Awais et al. [14]	Autofluorescence imaging	GLCM, LDA, KNN	HPIL system: 83% accuracy, 85% sensitivity, 84% specificity	83	85	84	Prospective technical validation study
12	Rahman et al. [15]	Histopathological images	Traditional ML	Used morphological and textural features for 11OSCC cla12ssification13	—	—	—	Retrospective observational diagnostic validation study
13	Das et al. [16]	Histopathological images	Transfer learning, CNN	Automated epithelial tissue cell classification	—	—	—	Experimental DL-based validation study
14	Rajan et al. [17]	Medical images	Deep neural network	Fog computing-based cancer classification system	—	—	—	Experimental DL-based validation in IoT/fog system
15	Jeyaraj et al. [18]	Medical images	Deep learning	Computer-aided image classification for early diagnosis	—	—	—	Experimental DL-based diagnostic validation
16	Zlotogorski-Hurvitz et al. [19]	Salivary exosomes	Computational analysis	FTIR with computational methods for diagnosis	—	—	—	Retrospective diagnostic validation study
17	Ariji et al. [20]	CT images	Deep learning	Assessed lymph node metastasis using AI	—	—	—	Retrospective diagnostic validation study
18	Al-Ma'aitah et al. [21]	Medical data	GSO-ESNN	Enhanced oral cancer detection model	—	—	—	Experimental deep learning validation study
19	Grillone et al. [22]	Elastic scattering spectroscopy	—	Margin guidance during surgery	—	—	—	Prospective analytic study
20	Liu et al. [23]	Clinical data	Quantitative prediction model	Predicted oral cancer risk in leukoplakia patients	—	—	—	Retrospective cohort risk prediction study
21	Abram et al. [24]	Cytology-on-a-chip	—	Chip-based monitoring of PMOLs	—	—	—	Proof-of-concept diagnostic feasibility study
22	Ye et al. [25]	Clinical images	Deep learning	Automated lesion detection	—	—	—	Prospective multicenter diagnostic validation study
23	Xie et al. [26]	Oral mucosal images	Self-attention, FDL	Oral mucosal disease recognition using advanced DL	—	—	—	Experimental DL-based validation study
24	Koriakina et al. [27]	Clinical images	Deep MIL	Interpretable AI vs traditional methods	—	—	—	Experimental DL-based validation study
25	Li et al. [28]	Raman spectroscopy	Deep learning	Multi-task detection using optical fiber Raman	—	—	—	Experimental DL-based validation study
26	Sukegawa et al. [29]	Histopathological images	Deep learning	Evaluated DL classifiers with pathologists	—	—	—	Experimental diagnostic validation study (histopathology)
27	Desai KM et al. [30]	Clinical images	Deep learning models (CNNs)	Demonstrated high diagnostic performance in classifying OPMDs and oral cancers from clinical images.	88.6	91.3	86.4	Prospective diagnostic validation study
28	Lepper TW et al. [31]	Cytopathology (AgNORs)	Deep learning models (CNNs)	Automated quantification of NORs using AI improved oral cancer screening reliability.	87	85.4	88.1	Retrospective diagnostic validation (cytopathology)

Discussion

The use of AI in healthcare has grown rapidly, with recent advancements demonstrating promising results in the early detection and diagnosis of OC. AI's strengths, including scalability, easy accessibility for screening, time efficiency, and improved diagnostic accuracy, make it especially valuable in high-burden and resource-constrained settings. These tools can work with a wide range of data, such as clinical photographs, pathology slides, radiographs, and molecular information. With rapid progress in this area, staying up to date with ongoing research is vital.

Considering the above facts, the present paper attempts to conduct a comprehensive review on the application of AI in the early detection of OC. An extensive literature search was conducted across major databases (PubMed), focusing on studies published between January 2016 and May 2025. In addition, we employed a comprehensive search strategy that combined MeSH terms, keywords, and manual reference checks. Considering the inclusion and exclusion criteria, 28 full-text original research articles were selected for detailed evaluation [4-31]. Notably, most of the studies originated from countries with a high prevalence of OC, particularly in South and Southeast Asia, highlighting the relevance and urgency of AI-driven approaches.

Table 2 summarizes the input data types, highlighting the diversity of data used to develop the models. The studies employed various approaches and utilized data from several sources, including clinical photos, smartphone images, radiographs, cytology samples, and saliva [4-29]. Recently, Desai et al. [30] showed that DL could effectively be used to screen for oral potentially malignant disorders and OC using a large set of photographic images. Their model performed well in terms of sensitivity and specificity, highlighting its potential use in community-based screening programs. Similarly, Lepper et al. [31] introduced AI for cytopathological quantification of nucleolar organizer regions (NORs), a key nuclear marker in OC. Their approach showed potential for integrating cytology-based AI tools into routine screening protocols, particularly in early detection contexts.

Among the AI techniques explored, convolutional neural networks (CNNs) or CNN-based architectures have consistently emerged as the most effective and widely used method [6,10,12,30]. CNN models are well-suited for image analysis and helpful in identifying oral lesions from photographs and radiographs. For example, Song et al. [6,12] applied CNNs for mobile and high-risk population screening. Warin et al. [10] achieved high diagnostic accuracy, with sensitivity and specificity values reaching 94% and 97% when analyzing photographic images. Desai et al. [30] further validated CNN-based models for detecting OPMDs and OC from clinical images, supporting their utility in mass screening and low-resource settings. DL models, particularly those based on CNNs, demonstrated high diagnostic performance in several studies, with reported accuracies reaching 91.4%, sensitivities up to 94%, and specificities up to 97% [6,10,12,16]. This indicates their strong potential for early and accurate detection of OPMDs and OCs.

Traditional AI techniques, such as artificial neural networks (ANNs), showed variable performance across studies. Alhazmi et al. [11] used ANN-based models and reported 78.95% accuracy, with sensitivity of 85.71% but specificity of only 60%, indicating modest diagnostic utility. Interestingly, Monedeiro et al. [13] found that when ANNs were used to analyze salivary volatile organic compounds (VOCs), the model achieved perfect sensitivity and specificity, along with 90% accuracy. This finding reinforces how much the type of data and the way a model is fine-tuned can influence diagnostic success.

DL algorithms, particularly convolutional neural networks (CNNs), have emerged as a significant trend in image-based diagnostics. Studies such as Lin et al. [8], Warin et al. [10], and Song et al. [12] applied CNNs to photographic images or smartphone-based captures, achieving high sensitivity and specificity in oral lesion classification. These models have shown real promise for use in low-resource settings and point-of-care environments, particularly in large-scale screening programs. Building on this, Desai et al. [30] used DL to automatically analyze clinical images and identify OPMDs and OC. The strong performance of their model suggests that it could be a valuable tool for broader public health initiatives.

Alternative AI-integrated imaging modalities have also shown promise. Caughlin et al. [5] used fluorescence lifetime imaging combined with end-to-end neural networks for in vivo diagnosis, while Narayanan et al. [9] used cloud-based multispectral imaging for real-time detection. These methods highlight the benefit of integrating AI with multimodal data to improve detection accuracy by mapping biochemical and morphological changes.

AI also holds potential in personalized diagnosis and prognostication. For instance, Kim et al. [4] employed subnetwork representation learning on transcriptomic data to predict lymph node metastasis. Rahman et al. [15] applied morphological and textural analysis using ML to stratify OC risk based on histological patterns. In cytology and histopathology, Das et al. [16] and Sukegawa et al. [29] demonstrated that CNNs and transfer learning could improve cell-level classification and assist in digital histopathological workflows. Extending this, Lepper et al. [31] used AI to quantitatively analyze nucleolar organizer regions (NORs) in cytological smears, offering a novel nuclear-level biomarker for early cancer screening.

Models leveraging salivary biomarkers and breath analysis introduce non-invasive, accessible avenues for early detection. Mentel et al. [7] and Monedeiro et al. [13] reported accurate predictions using VOCs, while Zlotogorski-Hurvitz et al. [19] used spectral analysis of salivary exosomes. This non-invasive approach could be constructive in spotting OC early, even before symptoms appear, giving hope for a future where early detection is more accessible and real.

Interestingly, researchers like Koriakina et al. [27] and Li et al. [28] have worked on making AI models more transparent and versatile, aiming to address the common concern that these systems often function as "black boxes" in clinical decision-making. Our findings support previous reviews by Garcia-Pola et al. [32] and Khanagar et al. [33], which focused on early developments in AI for OC. In addition, our review encompasses more recent studies that demonstrate improved diagnostic accuracy, integration into real-time tools, and diverse applications. This reflects the field’s ongoing progress and expanding clinical relevance.

CNN-based DL models dominate the current landscape due to their adaptability, strong performance, and scalability. There is a need for further innovation and validation for safe and effective clinical application of AI technologies for OC diagnostics, as traditional AI methods remain relevant, especially when matched with optimized data inputs. Although the studies were considerably heterogeneous in AI methods, data sources, and sample sizes, most reported promising performance outcomes.

Strengths and Limitations

Speed, accessibility, and cost-effectiveness have established AI as an emerging and highly effective early OC detection platform. Factors like applicability in low-resource settings, where mobile-based imaging facilitates earlier detection and timely referral, make AI very valuable. Desai et al.'s contribution [30] exemplifies this, as their approach can be used for large-scale screening in community settings with minimal infrastructure.

Some studies had limitations, such as using small or institution-specific datasets, a lack of external validations, and a lack of testing the model's generalization in diverse settings [11,14,17,21]. These concerns underline the need for consistent ways to develop, test, and share information about models, primarily when intended for clinical settings like chair-side screening, etc. Moreover, while the approach by Lepper et al. [31] shows potential, it needs further validation across broader populations to standardize NOR-based AI models in cytopathology.

A key limitation of this review is the exclusive use of PubMed for literature search. To address this, we employed detailed MeSH and keyword strategies, as well as manual reference checks. Future reviews should consider broader database inclusion.

While it holds much promise, AI can be prone to bias, which must be carefully addressed using diverse patient data. However, AI can complement clinicians to speed up early detection.

Recommendations and Future Directions

Healthcare professionals must test these tools in real-world settings to confirm they support clinical decision-making. Researchers must also gather more evidence to fully trust their performance in different clinical environments. There is a pressing need for large-scale, multicentric prospective studies, the development of ethical frameworks to ensure data privacy and reduce bias, the standardization of input formats and imaging protocols, and the creation of training programs to help clinicians integrate AI responsibly into practice.

## Conclusions

AI technologies are increasingly integrated into accessible tools such as smartphone apps, telehealth platforms, and portable diagnostic devices, making them particularly useful for community outreach and in areas where access to specialists is limited for the early detection and diagnosis of OPMDs and OCs.

 Working with oral pathologists, oncologists, dentists, public health experts, and policymakers can develop tools that are technically dependable, ethical, user-friendly, and aligned with clinical realities. When used effectively, AI can reduce delays in diagnosis and ultimately enhance patient outcomes. While challenges persist, such as ensuring fairness, minimizing bias, and earning the trust of both clinicians and patients, the path forward is promising.
